# Automated Classification of Colorectal Neoplasms in White-Light Colonoscopy Images via Deep Learning

**DOI:** 10.3390/jcm9051593

**Published:** 2020-05-24

**Authors:** Young Joo Yang, Bum-Joo Cho, Myung-Je Lee, Ju Han Kim, Hyun Lim, Chang Seok Bang, Hae Min Jeong, Ji Taek Hong, Gwang Ho Baik

**Affiliations:** 1 Department of Internal Medicine, Hallym University College of Medicine, Chuncheon 24252, Korea; yjyang@hallym.or.kr (Y.J.Y.); hlim77@hallym.or.kr (H.L.); cloudslove@hallym.or.kr (C.S.B.); cromnjeong@gmail.com (H.M.J.); hahahjt2@naver.com (J.T.H.); baikgh2@hanmail.net (G.H.B.); 2 Institute for Liver and Digestive Diseases, Hallym University, Chuncheon 24253, Korea; 3 Medical Artificial Intelligence Center, Hallym University Medical Center, Anyang 14068, Korea; mach.ai.leemj@gmail.com; 4 Division of Biomedical Informatics, Seoul National University Biomedical Informatics (SNUBI), Seoul National University College of Medicine, Seoul 03080, Korea; juhan@snu.ac.kr; 5 Department of Ophthalmology, Hallym University Sacred Heart Hospital, Anyang 14068, Korea

**Keywords:** colonoscopy, colorectal neoplasm, artificial intelligence, deep learning, convolutional neural network

## Abstract

**Background:** Classification of colorectal neoplasms during colonoscopic examination is important to avoid unnecessary endoscopic biopsy or resection. This study aimed to develop and validate deep learning models that automatically classify colorectal lesions histologically on white-light colonoscopy images. **Methods:** White-light colonoscopy images of colorectal lesions exhibiting pathological results were collected and classified into seven categories: stages T1-4 colorectal cancer (CRC), high-grade dysplasia (HGD), tubular adenoma (TA), and non-neoplasms. The images were then re-classified into four categories including advanced CRC, early CRC/HGD, TA, and non-neoplasms. Two convolutional neural network models were trained, and the performances were evaluated in an internal test dataset and an external validation dataset. **Results:** In total, 3828 images were collected from 1339 patients. The mean accuracies of ResNet-152 model for the seven-category and four-category classification were 60.2% and 67.3% in the internal test dataset, and 74.7% and 79.2% in the external validation dataset, respectively, including 240 images. In the external validation, ResNet-152 outperformed two endoscopists for four-category classification, and showed a higher mean area under the curve (AUC) for detecting TA+ lesions (0.818) compared to the worst-performing endoscopist. The mean AUC for detecting HGD+ lesions reached 0.876 by Inception-ResNet-v2. **Conclusions:** A deep learning model presented promising performance in classifying colorectal lesions on white-light colonoscopy images; this model could help endoscopists build optimal treatment strategies.

## 1. Introduction

Colorectal cancer (CRC) is one of the leading causes of death in the world, and the third most common malignancy in Korea [1,2]. Colonoscopy is an effective screening tool for the early identification and removal of colorectal neoplasms, and thus have been adopted in many countries to reduce the CRC mortality [3,4,5]. However, superfluous endoscopic resection or biopsy of diminutive non-neoplastic lesions increases unnecessary colorectal mucosal injury and medical economic burden [6,7,8]. On the contrary, underestimating colorectal cancers may lead to unsuitable treatment and the increase in mortality [4]. Therefore, it is essential to discriminate colorectal lesions accurately during colonoscopy, in order to choose an optimal treatment strategy.

For the optical diagnosis of colorectal lesions, several advanced endoscopic imaging technologies, including narrow-band imaging (NBI) with or without magnifying techniques, flexible spectral imaging color enhancement, or i-Scan digital contrast have been introduced and validated for efficacy [9,10,11]. Nevertheless, the diagnostic superiority of these advanced imaging technologies has not been proved in clinical practice, and the inter-observer and intra-observer variability were major hurdles to the generalization [12,13].

With the recent advancement of artificial intelligence (AI), automated diagnosis or classification of diseases on medical images such as retinal fundus photographs or clinical skin photos has been achieved in various fields of medicine [14,15], as well as in gastroenterology [16,17]. Some achieved promising performances in the field of colonoscopy, not only for the detection of colorectal polyps [18,19,20], but also for the classification of colorectal polyps [21,22,23]. However, most studies focused on the differentiation of neoplastic lesions from non-neoplastic lesions using specific images from magnifying NBI or endocytoscopy [21,22,24], which has limited the application in real-world practice. Therefore, this study aimed to develop and assess convolutional neural network (CNN) models which automatically categorize colorectal lesions into several stages ranging from non-neoplastic lesions to advanced CRC with conventional white-light colonoscopy images.

## 2. Materials and Methods

### 2.1. Data Collection

Patients who were diagnosed and treated for CRC between 2008 and 2017 were retrospectively included from three Hallym University-affiliated hospitals: Chuncheon Sacred Heart Hospital, Dongtan Sacred Heart Hospital, and Hallym University Sacred Heart Hospital. Static white-light colonoscopy images for any colorectal lesion of the patients were collected from the picture archiving and communication system (PACS) of the involved hospitals in a resolution of 640 × 480 pixels. When a patient had multiple colorectal lesions, each lesion was separately included. For one colorectal lesion, one or two representative images having different directions or distances to the object were selected. No annotations for the images were extracted from the PACS for training. The exclusion criteria were as follows: (1) blurred or defocused images, (2) images from patients with inflammatory bowel disease, infectious colitis, ischemic colitis, or poor bowel preparation, (3) images from chromoendoscopy or image-enhanced endoscopies including NBI, (4) images of colorectal lesions without pathologic results, (5) images of sessile serrated adenoma/polyps, or traditional serrated adenomas. Ultimately, 3828 white-light colonoscopy images from 1339 subjects were involved in this study, apart from the external validation dataset. This study was approved by the Institutional Review Board of Chuncheon Sacred Heart Hospital (IRB No. 2018-05) and was performed in accordance with the Declaration of Helsinki.

### 2.2. Colonoscopy Procedure

Colonoscopy examinations at the three Hallym University-affiliated hospitals were performed by expert faculty doctors having experience with >2000 cases of colonoscopy, after bowel preparation using 4 L of polyethylene glycol or 2 L of polyethylene glycol with ascorbic acid. The colonoscopy equipment models used in this study were CF-HQ290L/I, CF-H290L/I, CF-Q260AL/I, and CF-H260AL (Olympus Optical Co., Ltd., Tokyo, Japan) and EC-600WI, EC-590WM, EC-590ZW, EC-600WM, EC-760R-VM, and EC-760ZP-VM (Fujinon Co., Saitama, Japan). All the colorectal lesions were entirely removed by forceps biopsy, endoscopic mucosal resection, endoscopic submucosal dissection (ESD), or surgical resection. The final pathologic results of the colorectal lesions in the collected images were all identified and used as the reference standard. The pathologic results were (1) adenocarcinoma for CRC, (2) tubular adenoma with high grade dysplasia, (3) tubular adenoma with or without low grade dysplasia, and (4) hyperplastic polyps, inflammatory polyps, lymphoid polyps, chronic inflammation, leiomyoma, lipoma for non-neoplastic lesions.

### 2.3. Data Classification

All the static white-light colonoscopy images were reviewed by an experienced colonoscopist (Y.J.Y.) and were identified as one of seven categories according to the final pathologic results: CRC (stages T1, T2, T3, and T4), high-grade dysplasia (HGD), tubular adenoma with or without low grade dysplasia (TA), and non-neoplastic lesions. HGD included not only tubular adenoma with high-grade dysplasia but also carcinoma in situ and intramucosal cancer. The non-neoplastic lesions included any form of hyperplastic polyps, inflammatory polyps, lymphoid polyps, chronic inflammation, and submucosal tumors except neuroendocrine tumor. When the colonoscopy images and the pathological readings were reviewed, the gross findings of colorectal lesions were consistent with the pathologic results of the corresponding lesions in almost all cases of the original dataset. Ultimately, among 3828 images, the majority class was TA lesions (1316 cases, 34.4%), followed by non-neoplastic lesions (896 cases, 23.4%), and HGD (621 cases, 16.2%) (Supplementary Table S1).

Next, the images from the seven categories were reclassified into four categories combining similar categories into one class: advanced CRC (stages T2, T3, and T4), early CRC/HGD (stage T1 and HGD), TA, and non-neoplastic lesions. Apart from the above 4-category classification, the images were also categorized into two groups: advanced colorectal lesions (HGD and T1, T2, T3, and T4 lesions) and non-advanced colorectal lesions (TA and non-neoplastic lesions). Finally, the images were also categorized into two groups from another standpoint: neoplastic lesions (TA, HGD, and stages T1, T2, T3, and T4) vs. non-neoplastic lesions.

### 2.4. The Training, Test and External Validation Datasets

To build the classification models, the patients in the original dataset were divided into a training dataset and a test dataset at ratio of 9:1 using random sampling in each lesion category. Random sampling was performed using the patient ID number as the key, to prevent the same class photographs of the same patient from entering into both the train and test datasets simultaneously and thus to avoid the overestimation of the model performance [17]. The training dataset and the test dataset were mutually exclusive and collectively exhaustive for the whole original dataset.

**Figure 1:**
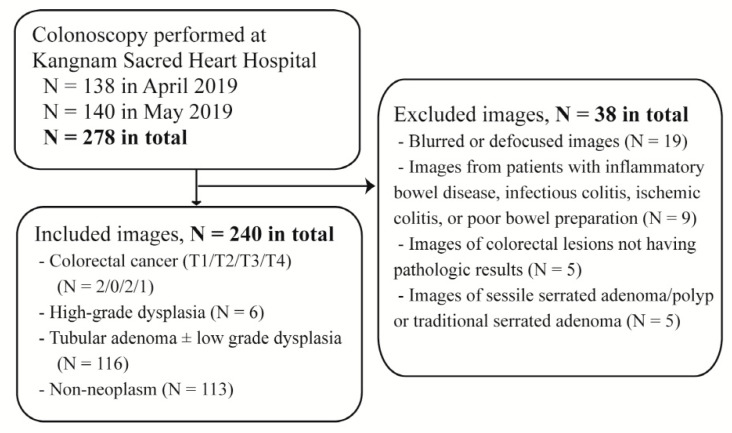
Flow diagram of the external validation procedure.

In addition, an external validation dataset was constructed including patients from another hospital that did not participate in the collection of the original dataset. The static white-light colonoscopy images were collected from the consecutive patients who underwent a colonoscopy for various reasons at Kangnam Sacred Heart Hospital between April and May in 2019 (Figure 1). The exclusion criteria were as same as mentioned above. The colonoscopy examinations were performed for routine health check-ups, diagnosis of gastrointestinal symptoms, or treatment of neoplastic lesions. Of note, the external validation dataset included only one representative image of the histologically most advanced lesion from each patient. For example, if a patient had one tubular adenoma and one hyperplastic polyp, only one representative image of the tubular adenoma was included. Finally, 240 images from 240 patients were included in the external validation dataset, which comprised 3 images of advanced CRC, 8 images of early CRC/HGD, 116 images of TA, and 113 images of non-neoplastic lesions. Furthermore, of the 240 patients, 161 (67.1%) were male, and the mean age was 63 years (SD, 12.1 years).

### 2.5. Preprocessing of the Datasets

To increase the model performance, data augmentation was performed using horizontal flipping and/or vertical flipping, amplifying the dataset four times. Afterwards, the images were normalized for each RGB color channel.

### 2.6. Construction of the CNN Models

To construct the deep learning models, two CNN architectures, namely, ResNet-152 and Inception-ResNet-v2, were used. The models were pre-trained using the ImageNet Large Scale Visual Recognition Challenge dataset. The details of the CNN architectures are described elsewhere [25,26].

The training process consisted of three stages, increasing the resolution of images from 400 × 400 to 450 × 450 pixels, and finally making the resolution 480 × 480 pixels. In each stage, the last few layers were unfrozen first and trained, and then the entire layers were unfrozen and trained using different learning rates for the first few, middle, and last layers.

In each training step, cyclic learning suggested by Huang et al. was used with cosine annealing with stochastic gradient descent, but without adopting a snapshot ensemble [27]. The initial learning rate was 1e-3, and the number of cycles was four. The initial cycle length was one, and the cycle length multiplier was four. Early stopping was used to minimize the validation loss in one cycle.

To provide the region of interest, a class activation map (CAM) was implemented with a continuous color spectrum. The last two layers of the CNN models were removed, and global average pooling and softmax layers were added. The dropout rate was 0.5, and the batch size was 6. All training was performed using NVIDIA GeForce GTX 1080ti graphics processing units with dual Xeon central processing units and 128 GB RAM, based on the PyTorch platform.

### 2.7. Main Outcome Measures

The performances of the established CNN models were assessed using the test dataset and the external validation dataset. Each prediction was based on test-time augmentations (TTA) using the original, horizontally flipped, vertically flipped, and horizontally vertically flipped images. To minimize the bias from random sampling the test dataset, a random split of the training/test dataset was performed three times, and the mean accuracy was evaluated.

The primary outcomes were the discrimination performance of the established CNN models for seven-category classification and four-category classification. The secondary outcomes were binary discrimination performance of the established CNN for advanced colorectal lesions vs. non-advanced colorectal lesions, and colorectal neoplastic lesions vs. non-neoplastic lesions.

For external validation, two endoscopists (C.S.B. and J.T.H.) with more than 5 years of colonoscopy experience each and 1 novice endoscopist (H.M.J.) in the fellowship course in gastroenterology with 6 months of colonoscopy experience were asked to categorize the static white-light colonoscopy images in the external validation dataset into four classes without knowing the pathologic results. They were all from Chuncheon Sacred Heart Hospital which is different from the hospital where the external validation dataset was collected (Kangnam Sacred Heart Hospital). They evaluated the colonoscopy images only for the purpose of the external validation of the present study. The number of annual colonoscopies was about 900 and 1000 for the two experienced endoscopists, respectively. The novice endoscopist performed about 260 full colonoscopy examinations without assistance of supervisor until external validation test. The adenoma detection rate (ADR) of two experienced endoscopists was 40.4% and 44.4%. The discrimination performances of these three endoscopists were compared to those of the constructed CNN models.

### 2.8. Statistical Analysis

To evaluate the ability of the established CNN models, the area under the receiver operating characteristic (ROC) curve (AUC) was estimated. In addition, the diagnostic accuracy, sensitivity, specificity, positive predictive value, and negative predictive value were calculated. Continuous or categorical variables were expressed as means or percentages with 95% confidence intervals (CIs), respectively. Categorical variables were compared with Fisher’s exact test, and the AUC values were compared with the DeLong test. A two-tailed *p* value <0.05 was considered statistically significant in this study. All of the analyses were performed using SPSS version 24.0 (SPSS Inc., Chicago, IL, USA) and MedCalc version 19.1 (MedCalc Software, Ostend, Belgium).

## 3. Results

### 3.1. Seven-Class Classification Performances

**Figure 2:**
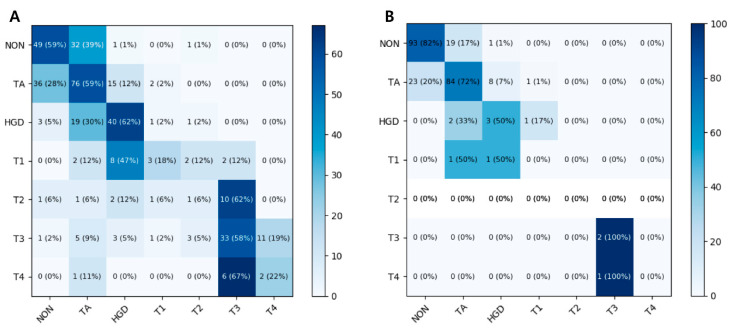
Heat maps of the ResNet-152 confusion matrices for seven-category classification scheme for colorectal lesions on colonoscopic photographs (**A**) in the test dataset and (**B**) in the external validation dataset. NON, non-neoplastic lesion; TA, tubular adenoma; HGD, high-grade dysplasia.

The mean accuracy for the seven-class classification in the test dataset was 60.2% (95% CI 55.9–64.5%) by the ResNet-152 model and 56.4% (95% CI 53.8–59.0%) by the Inception-ResNet-v2 model. The mean accuracy of the CNN model in the external validation dataset was 74.7% (95% CI 73.3–76.2%) by the ResNet-152 model and 74.3% (95% CI 72.1–76.5%) by the Inception-ResNet-v2 model. A heat map of the confusion matrix of the best-performing model (ResNet-152) is shown in Figure 2. The per-class accuracy was highest for HGD (62%) in the test dataset and for T3 (100%) in the external validation dataset.

### 3.2. Four-Class Classification Performances

**Figure 3:**
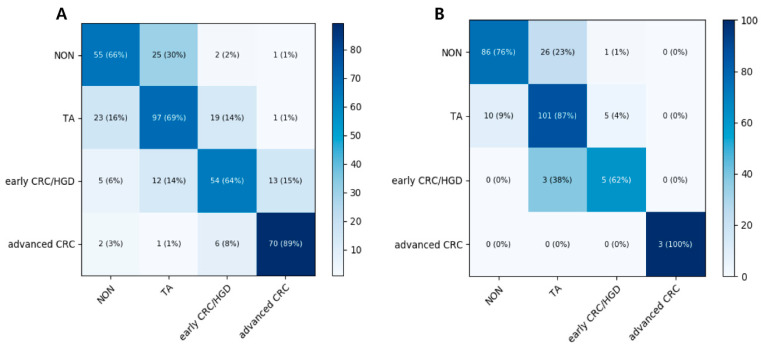
Heat maps of the ResNet-152 confusion matrices for four-category classification scheme for colorectal lesions on colonoscopic photographs (**A**) in the test dataset and (**B**) in the external validation dataset. NON, non-neoplastic lesion; TA, tubular adenoma; HGD, high-grade dysplasia; CRC, colorectal cancer.

The mean accuracy for the four-category prediction in the test dataset reached 67.3% (95% CI 62.7–71.8%) by the ResNet-152 model, and 67.7% (95% CI 63.2–72.1%) by the Inception-ResNet-v2 model. The mean accuracy of the CNN model in the external validation dataset was 79.2% (95% CI 76.5–81.8%) by the ResNet-152 model and 76.0% (95% CI 73.6–78.4%) by the Inception-ResNet-v2 model. A heat map of the confusion matrix of the better-performing model (ResNet-152) is shown in Figure 3. Advanced CRC and TA showed good accuracy in both test and external validation dataset.

When three endoscopists categorized the external validation dataset into four classes, the accuracies of the best- and worst-performing endoscopist were 85.5% (95% CI 81.0–89.9%) and 68.0% (95% CI 62.1–73.9%), respectively. The detailed per-class performance summary of endoscopists and the CNN models for the four-class classification of colorectal lesions is presented in Supplementary Figure S1. There was no significant difference in the performances of the best-performing endoscopist and ResNet-152 (*p* = 0.46). However, the performance of ResNet-152 was significantly higher than those of the two other endoscopists (*p* < 0.001, and 0.04, respectively).

### 3.3. Binary Classification Performances

**Figure 4:**
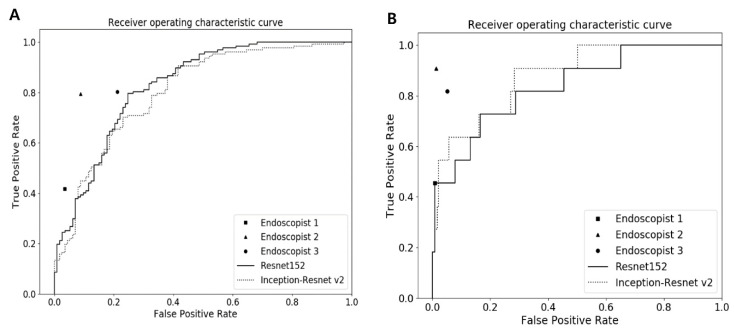
Receiver operating characteristic curves for binary classification of (**A**) colorectal neoplasms (TA or higher) and (**B**) advanced colorectal lesions (HGD or higher) in the external validation dataset. TA, tubular adenoma; HGD, high-grade dysplasia.

The detailed diagnostic performances of the CNN models and human endoscopists for binary classification in the test dataset are presented in Table 1. For the binary classification for neoplastic (TA or higher grade) vs. non-neoplastic lesions in the test dataset, the mean AUC and the accuracy of the better-performing model (Inception-ResNet-v2) was 0.832 (95% CI 0.810–0.854) and 79.5% (95% CI 77.6–81.4%), respectively. For external validation dataset, the mean AUC of Inception-ResNet-v2 (AUC 0.760 (95% CI 0.753–0.767)) was significantly higher than that of the worst-performing endoscopist (AUC 0.691 (95% CI 0.628–0.749), *p* < 0.001), but significantly lower than that of the best-performing endoscopist (AUC 0.853 (95% CI 0.802–0.896), *p* < 0.001) (Figure 4 and Supplementary Table S2). And the accuracy of Inception-ResNet-v2 in external validation dataset was 71.5% (95% CI 68.0–75.0%) similar to that of the worst-performing endoscopist (67.5% (95% CI 61.2–73.9%), *p* = 0.37).

The binary classification performances for advanced colorectal lesions (HGD or higher grade) vs. non-advanced colorectal lesions in the test dataset, the Inception-ResNet-v2 model, showed better performances with AUC of 0.935 (95% CI 0.929–0.941) and accuracy of 87.1% (95% CI 86.2–88.0%) than those of ResNet-152. For external validation dataset, all the endoscopists and CNN models showed high accuracy of more than 90% (Supplementary Table S2). The Inception-ResNet-v2 (94.6% (95% CI 93.8–95.4%)) achieved similar accuracy to the two residual endoscopists (96.7% (95% CI 93.5–98.6%) and 94.2% (95% CI 90.4–96.8%)) excluding the best-performing endoscopist. The AUC of the Inception-ResNet-v2 (AUC 0.876 (95% CI 0.873–0.879)) was higher than the AUC of the worst performing endoscopist with a marginal significance (AUC 0.723 (95% CI 0.662–0.779), *p* = 0.05).

### 3.4. Class Activation Map

**Figure 5:**
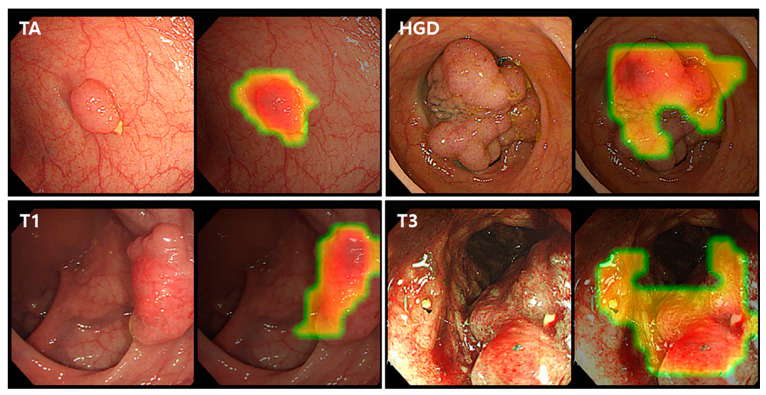
Class activation maps of the binary classification model detecting neoplasms (TA or higher) for several colorectal lesions. TA, tubular adenoma; HGD, high-grade dysplasia.

Representative CAM images of the best performing Resnet-152 model for binary classification detecting neoplastic lesions (TA or higher) are shown in Figure 5. The CNN model detected neoplastic lesions well in the colonoscopy images. For TA and HGD lesions, the CAM localized the elevated polyp lesions as the regions of interest. For the identification of CRC including T1 and T3, the CAM suggested depressions or ulcerations with mucosal irregularities or spontaneous bleeding areas as the regions of interest.

## 4. Discussion

This study showed the promising performance of deep learning models in differentiating colorectal lesions into multiclass categories as well as into binary categories. To the best of our knowledge, this is the first study to use CNN models to classify the full spectrum of colorectal neoplasms using static white-light colonoscopy images. Our CNN models have an advantage in that we extended the discrimination ability for advanced colorectal lesions, including HGD and CRC, as well as non-neoplastic lesions, which could guide the selection of an optimal treatment strategy for endoscopists. Additionally, as conventional white-light colonoscopy images were used, our CNN models could be easily adopted in current clinical practice without additional training. In addition, we used colonoscopy images from multiple centers using various kinds of colonoscopes, which facilitated the generalizability of our CNN models regardless of the type of colonoscope. Finally, we also compared the performances of the established CNN models with those of three endoscopists. Our CNN models showed better or similar performances than those of the worst-performing endoscopists in the external validation, which supported the idea that the application of our CNN models could assist endoscopists in the discrimination of colorectal lesions.

Accurate and reliable optical diagnosis of colorectal lesions, which is essential to determine the best therapeutic strategy, has been challenged in clinical practice. Although several image-enhanced endoscopic techniques have been applied in clinical practice with various classification systems using NBI (such as NBI international colorectal endoscopic and Japanese NBI expert team classification to enhance the characterization of colorectal lesions [10,28,29]), previous studies showed that training and experience are prerequisites for achieving the accurate prediction of an optical diagnosis using advanced imaging technologies [12,13]. There were also concerns about the effect of inter-observer and intra-observer variability on interpretation and the inaccessibility in adopting these advanced technologies in the real world. To overcome these problems, various working groups have developed computer-aided diagnosis (CAD) systems to improve the characterization of colorectal lesions [21,22,23,30,31]. In early studies, the CAD system usually used the features extracted by human efforts. However, with the technical progress, deep neural networks (especially CNN) were involved in both feature extraction and classification, thus enhancing diagnostic accuracy [31]. Zhang et al. demonstrated better performance of a novel transfer learning algorithm using deep CNN than that of endoscopists in terms of recall rate (87.6% vs. 77.0%), and accuracy (85.9% vs. 74.3%) for the automatic detection and classification of colorectal polyps [30]. However, studies that explored a CAD system for the histologic prediction of colorectal lesions beyond hyperplastic polyps are currently limited [32,33,34]. Although Takeda et al. reported the substantial performance of a CAD system in distinguishing adenoma from invasive cancer, they used images from ultrahigh magnification images with an integrated-type endocytoscope, which is rarely available in current practice [33]. Recently, Sanchez-Montes et al. reported on a CAD system to predict colorectal polyp histology with white-light colonoscopy images [34]. However, they solely classified dysplastic and non-dysplastic groups using support vector machines, which guided the identification of dysplastic lesions with surface patterns. On the other hand, we developed a CNN model, which automatically classifies the full spectrum of colorectal lesions, and we divided the prediction categories to identify HGD (including carcinoma in situ and intramucosal cancer) and early CRC because they are crucial candidates for complete endoscopic resection. Our CNN model showed promising performance in the four-category and binary classification schemes for detecting advanced colorectal lesions; the model could be used to avoid inappropriate invasive interventions such as ESD or surgical resection and intervention-related adverse events in cases of tubular adenoma or non-neoplastic lesions by differentiating them from HGD and early CRC and in terms of reducing unnecessary endoscopic treatment in cases of advanced CRC by differentiating them from HGD and early CRC. Additionally, our CNN model led to more careful and meticulous inspection to determine the best treatment strategy or to assist in referring to high-volume, specialized hospitals for complete resection in the case of suspicious HGD or early CRC.

It is well known that non-neoplastic lesions have little potential for malignancy; resection-and-discard strategy or diagnose-and-leave strategy for diminutive (≤5 mm) hyperplastic polyps in the rectosigmoid colon, is suggested to save on costs associated with resection procedures and pathologic evaluations [35]. Therefore, several previous studies reported CNN models for the differentiation of neoplastic polyps from hyperplastic polyps. Byrne et al. proposed CNN models with an accuracy of 94% for binary classification of adenomatous polyps and hyperplastic polyps [21]. Chen et al. demonstrated a CAD system with an accuracy of 90.1%, a sensitivity of 96.3% and a specificity of 78.1% to identify neoplastic polyps including adenoma with HGD [22]. Although these studies showed higher diagnostic performance than that of our CNN model for binary classification of neoplastic and non-neoplastic lesion, they used the NBI images with or without magnifying, or chromoendoscopy images, while our CAD system only used white-light colonoscopy images. Moreover, our dataset included heterogeneous diseases in the non-neoplastic group involving not only hyperplastic polyps but also inflammatory polyp, lymphoid polyp, and chronic inflammation, and this could have influenced the performance of our models.

There were several limitations to be addressed in our study. First, the training and test datasets were collected retrospectively, and the training data were collected from the patients who were diagnosed with CRC, which could lead to selection bias. However, because the incidence of malignant colorectal lesions was low compared to the incidence of benign colorectal lesions in routine colonoscopy examinations, it is impossible to collect all colonoscopy images of benign neoplasms during the study period, when the colonoscopy images of malignant neoplasms are also sufficient to train the CNN models. Therefore, we had to collect colonoscopy images from patients with malignant colorectal lesions. To overcome this issue, we used all the colonoscopy images from consecutive patients regardless of the reason for examination as an external validation dataset. The prevalence rates of overall and advanced adenoma from the external validation dataset were comparable to those from colonoscopy screenings in individuals over fifty years old in Korea [36]. Second, we excluded images of sessile serrated adenoma/polyps and traditional serrated adenomas because the pathologic standard of these lesions is currently inconsistent, and there was a possibility of inter-observer variations in the histologic interpretation. However, because there is substantial malignancy potential in serrated adenoma/polyps, further study of the CNN model to distinguish serrated colorectal lesions is warranted. Third, our CNN models cannot be trained to differentiate superficial submucosal invasion from deep submucosal invasion of CRC because the white-light colonoscopy images of T1 CRC were too small in number and there was no mention of the invasion depth in the surgically resected specimens. However, distinguishing superficial submucosal invasion in cancer from deep submucosal invasion in cancer is difficult even for well-trained and experienced endoscopists, but it is an important ability to determine resectable T1 CRC in clinical practice. Therefore, a CAD system from a qualified, large number of T1 CRC datasets will be required to identify optimal candidates for endoscopic resection.

Nevertheless, our CNN models provided a quite useful classification performance with class activation maps enabling human endoscopists to see the region of interest. The first purpose of this deep learning model was to assist endoscopists to classify colorectal lesions found during colonoscopy examination on site by suggesting a secondary opinion. Endoscopists need to classify colorectal lesions on site to determine treatment strategy, but the decision should be fast and mostly depends on the experience of each endoscopist. This model would provide an additional opinion and help to reduce the misdiagnosis rate. If this model is applied to the real-time colonoscopy examination videos, it would help to reduce superfluous resection and to increase the sensitivity for defection of early lesions in the clinical field. Therefore, another purpose of this algorithm may be to help endoscopists to not miss small or negligible lesions in the examination, and possibly to analyze the videos automatically which were recorded by a capsule endoscopy system. To meet this need, a randomized clinical trial would be required in the future for the sound verification of this model.

In conclusion, our established CNN model showed promising performance in classifying colorectal neoplasms from non-neoplastic lesions to advanced CRC based on standard white-light colonoscopy images. Our CNN model could be adopted to assist in the accurate prediction of histology and in the choice of the best therapeutic strategy in real-world practice.

## Figures and Tables

**Table 1:** Diagnostic performance of deep-learning models for binary classification of colorectal lesions on colonoscopic photographs in the test dataset.

Model	Diagnostic Performance,% (95% CI)					AUC (95% CI)
	Accuracy (%)	Sensitivity (%)	Specificity (%)	PPV (%)	NPV (%)	
**Neoplastic lesions vs. non-neoplastic lesions**						
ResNet-152	79.4 (78.5–80.3)	95.4 (93.2–97.6)	30.1 (25.5–34.7)	80.8 (78.4–83.2)	68.8 (58.4–79.2)	0.821 (0.802–0.840)
Inception-ResNet-v2	79.5 (77.6–81.4)	94.1 (92.5–95.7)	34.1 (28.1–40.1)	81.6 (80.6–82.6)	65.0 (54.7–75.3)	0.832 (0.810–0.854)
**Advanced colorectal lesions vs. non-advanced colorectal lesions**						
ResNet-152	86.7 (84.9–88.5)	80.0 (75.4–84.6)	91.3 (90.8–91.8)	86.0 (83.7–88.3)	87.1 (85.1–89.1)	0.929 (0.927–0.931)
Inception-ResNet-v2	87.1 (86.2–88.0)	83.2 (81.5–84.9)	89.7 (87.7–91.7)	84.5 (81.0–88.0)	88.7 (87.7–89.7)	0.935 (0.929–0.941)

CI, confidence interval; PPV, positive predictive value; NPV, negative predictive value; AUC, area under the curve.

## Supplementary Materials

The following are available online at https://www.mdpi.com/2077-0383/9/5/1593/s1, Supplementary Table S1. Data composition of enrolled colonoscopic photographs in each dataset Supplementary Table S2. Diagnostic performance of three endoscopists and deep-learning models for binary classification of colorectal lesions on colonoscopic photographs in the external validation dataset Supplementary Figure S1. Per-class diagnostic performances of three endoscopists and deep-learning models for four-class classifications of colorectal lesions in the external validation dataset as for (A) advanced CRC or early CRC/HGD and (B) TA or non-neoplastic lesions. CRC, colorectal cancer; HGD, high-grade dysplasia; TA, tubular adenoma.
