# Supplementary material for: Automated Classification of Colorectal Neoplasms in White-Light Colonoscopy Images via Deep Learning
Source: J Clin Med. 2020 May 24;9(5):1593. doi: 10.3390/jcm9051593 (PMC7291169; doi:10.3390/jcm9051593)
Supplement: Supplementary file 1 [file jcm-09-01593-s001.zip › Supplementary_Table_1.docx]

**Supplementary Table 1**. Data composition of enrolled colonoscopic photographs in each dataset

|  | Whole Dataset | | Training set | | Test set | |
| --- | --- | --- | --- | --- | --- | --- |
|  | Image N | Patient N | Image N | Patient N | Image N | Patient N |
| Overall | 3828 | 1339 | 3453 | 1446 | 375 | 215 |
| **Seven-category classification** |  |  |  |  |  |  |
| Non-neoplastic lesion | 896 | 446 | 813 | 401 | 83 | 45 |
| TA | 1316 | 616 | 1187 | 554 | 129 | 62 |
| HGD | 621 | 476 | 557 | 428 | 64 | 48 |
| T1 | 185 | 142 | 168 | 128 | 17 | 14 |
| T2 | 141 | 93 | 125 | 93 | 16 | 10 |
| T3 | 591 | 424 | 534 | 382 | 57 | 42 |
| T4 | 78 | 48 | 69 | 43 | 9 | 5 |
| **Four-category classification** |  |  |  |  |  |  |
| Non-neoplastic lesion | 896 | 446 | 813 | 401 | 83 | 45 |
| TA | 1316 | 616 | 1176 | 554 | 140 | 62 |
| early CRC/HGD | 806 | 607 | 722 | 546 | 84 | 61 |
| Advanced CRC | 810 | 572 | 731 | 515 | 79 | 57 |

TA, tubular adenoma; HGD, high grade dysplasia; CRC, colorectal cancer.
