# Supplementary material for: Automated Classification of Colorectal Neoplasms in White-Light Colonoscopy Images via Deep Learning
Source: J Clin Med. 2020 May 24;9(5):1593. doi: 10.3390/jcm9051593 (PMC7291169; doi:10.3390/jcm9051593)
Supplement: Supplementary file 1 [file jcm-09-01593-s001.zip › Supplementary_Table_2.docx]

**Supplementary Table 2.** Diagnostic performance of three endoscopists and deep-learning models for binary classification of colorectal lesions on colonoscopic photographs in the external validation dataset.

| **Model** | **Diagnostic performance,% (95% CI)** | | | | | **AUC (95% CI)** |
| --- | --- | --- | --- | --- | --- | --- |
|  | **Accuracy (%)** | **Sensitivity (%)** | **Specificity (%)** | **PPV (%)** | **NPV (%)** |  |
| **Neoplastic lesions vs. non-neoplastic lesions** | | | | | | |
| Endoscopist 1 | 67.5 (61.2-73.9) | 41.7 (33.1-50.8) | 96.5 (91.2-99.0) | 93.0 (83.2-97.3) | 59.6 (55.9-63.2) | 0.691 (0.628-0.749) |
| Endoscopist 2 | 85.0 (79.8-89.3) | 79.5 (71.5-86.7) | 91.2 (84.3-95.7) | 91.0 (84.7-94.8) | 79.8 (73.1-84.9) | 0.853 (0.802-0.896) |
| Endoscopist 3 | 79.6 (73.9-84.5) | 80.3 (72.3-86.8) | 78.8 (70.1-85.9) | 81.0 (74.7-86.0) | 78.1 (71.2-83.7) | 0.791 (0.734-0.841) |
| ResNet-152 | 73.3 (68.8-77.8) | 91.9 (87.8-96.0) | 52.5 (38.2-66.8) | 68.8 (63.6-74.0) | 85.8 (82.1-89.5) | 0.818 (0.804-0.832) |
| Inception-ResNet-v2 | 71.5 (68.0-75.0) | 92.4 (92.4-94.4) | 44.2 (29.0-59.4) | 65.4 (59.7-71.1) | 83.6 (81.9-85.3) | 0.760 (0.753-0.767) |
| **Advanced colorectal lesions vs. non-advanced colorectal lesions** | | | | | | |
| Endoscopist 1 | 96.7 (93.5-98.6) | 45.5 (16.8-76.6) | 99.1 (96.9-99.9) | 71.4 (35.3-92.0) | 97.4 (95.7-98.5) | 0.723 (0.662-0.779) |
| Endoscopist 2 | 98.8 (96.4-99.7) | 90.9 (58.7-99.8) | 99.1 (96.9-99.9) | 83.3 (55.4-95.3) | 99.6 (97.2-99.9) | 0.950 (0.915-0.974) |
| Endoscopist 3 | 94.2 (90.4-96.8) | 81.8 (48.2-97.7) | 94.8 (91.0-97.3) | 42.9 (28.8-58.2) | 99.1 (96.9-99.7) | 0.883 (0.835-0.921) |
| ResNet-152 | 93.6 (92.1-95.1) | 48.5 (42.6-54.4) | 95.8 (94.2-97.4) | 36.7 (27.5-45.9) | 97.5 (97.3-97.7) | 0.829 (0.828-0.830) |
| Inception-ResNet-v2 | 94.6 (93.8-95.4) | 51.5 (45.6-57.4) | 96.7 (95.7-97.7) | 43.0 (37.6-48.4) | 97.6 (97.4-97.8) | 0.876 (0.873-0.879) |

CI, confidence interval; PPV, positive predictive value; NPV, negative predictive value; AUC, area under the curve
