# Supplementary figures and images for: Automated Classification of Colorectal Neoplasms in White-Light Colonoscopy Images via Deep Learning
Source: J Clin Med. 2020 May 24;9(5):1593. doi: 10.3390/jcm9051593 (PMC7291169; doi:10.3390/jcm9051593)

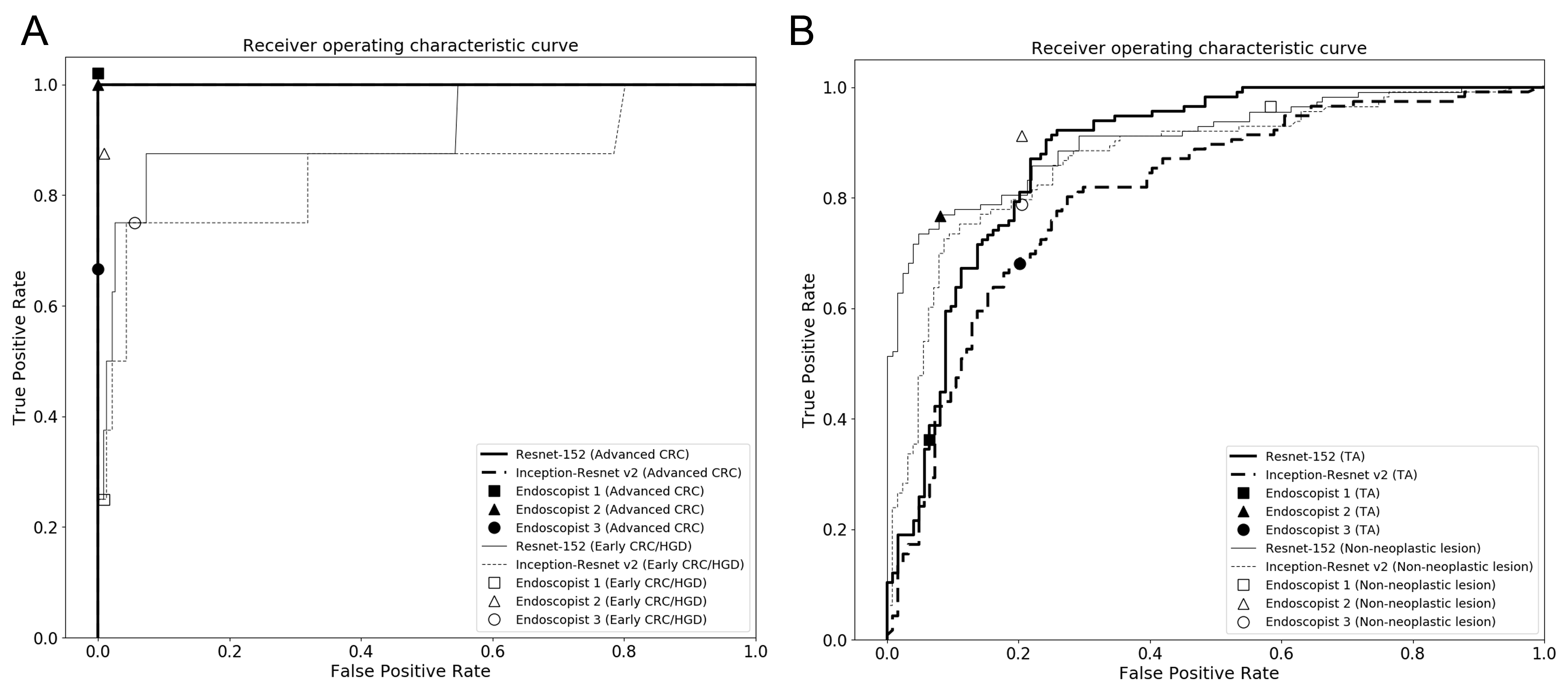

Supplement: Supplementary file 1 [file jcm-09-01593-s001.zip › Supplementary_Figure_1.tif]
